# Medical Facilities for Refugees in Europe: Creating a Consultation for Resettled Syrian Families

**DOI:** 10.3389/fmed.2021.728878

**Published:** 2021-11-26

**Authors:** Nahema El Ghaziri, Jeremie Blaser, Mary Malebranche, Brigitte Pahud-Vermeulen, Teresa Gyuriga, Joan-Carles Suris, Mario Gehri, Patrick Bodenmann

**Affiliations:** ^1^Department of Vulnerabilities and Social Medicine, University Center of General Medicine and Public Health, Lausanne, Switzerland; ^2^Department of Medicine, Dalhousie University, Halifax, NS, Canada; ^3^Department Woman-Mother-Child, Lausanne University Hospital, Lausanne, Switzerland

**Keywords:** refugees and asylum seekers, Syrian crisis, family consultation, interdisciplinary projects, innovation

## Abstract

The wave of migration that has hit Europe in recent years has led to several changes in the organization of asylum systems and medical care provided to migrants. Previous studies indicate that asylum seekers and refugees face multiple barriers in accessing health care. For that reason, adapted structures are needed. In this context, a family consultation service was implemented in our medical center in Lausanne, Switzerland. It aimed at addressing the unique health care needs of recently resettled families from Syria, which has been the leading source country for refugees since 2014. This intervention, developed through collaboration between the University Center for Primary Care and Public Health (Unisanté) and the Children's Hospital of Lausanne (HEL) involved a multidisciplinary team comprising a pediatrician, a general practitioner and a pediatric nurse. Bringing together a multidisciplinary team optimized care coordination, facilitated communication between care providers and enabled a more global vision of the family system with the aim of enhancing quality of care.

## Introduction

Managing refugees is a persistent and rising subject on the agenda of European countries. During the last decade, multiple political crises arose in a short period, such as the anti-government protests that led to the Arab spring. Simultaneously a restriction of the regular migration policies was adopted in many European Union (EU) countries (1). The combination of both aspects led to a significant inflation of asylum seekers. Accordingly, the number of non-EU asylum seekers applying for the first time across Europe started to significantly increase in 2011 until a peak was reached in 2015 with more than 1.25 million first-time applicants (2). Among these applications, 3% were done in Switzerland, which represented 4.9 applications per 1,000 habitants and was above the European numbers that were around 2.6‰. However, it should be noted that there were important disparities between European countries. For example, in Sweden the rate was of 16.6‰, in Austria of 10.2‰ and in Germany 5.3‰ (3). A more detailed and actual international comparison of the numbers of refugees and asylum seekers, as well as some commentaries from the Migration Policy Index 2020 (4) is added in Annex 1.

European countries have always been actively involved in the hosting of refugees. However, they were not prepared for the massive influx of 2015, which necessitated considerable adaptations for many governmental systems (5, 6) and encouraged innovation.

One of the services that appeared to require restructuring was (and still is) the management of the refugees' health needs. While refugees frequently arrive in a satisfying level of fitness, they appear to show a decline in health after a certain time in the hosting country (7). This reported decline in health may be explained by the fact that refugees face high challenges and are vulnerable to a number of threats to their health. We also know that restrictive entry and integration policies are linked to poor migrant health outcomes in high-income countries (8). As highlighted by Rechel (7), refugees' specific needs tend to be poorly understood. Newly arriving refugees frequently have different health necessities (both mental and physical), compared to nationals. Furthermore, the diseases they have been exposed to in their country of origin and during migration trajectories may not be familiar to the practitioners of the country of refuge. Additionally, language and cultural differences, as well as the lack of experience or even the fear concerning health services can prejudice the quality of delivered care (9). Even for refugees who were familiar with health services in their country of origin, understanding and adapting to a new health system is not easy. Particularly for families, as they have to navigate the care systems for both children and adults. Accordingly, appropriate communication is particularly important for this population, but is a recurrent issue as health systems are often insufficiently trained nor organized to respond effectively to the needs of those patients.

The consequence of all these aspects is that refugees face multiple barriers on their path to accessing adequate health care (10). Providing adjusted facilities may enhance equity and quality of care, while reducing health costs. For example, ensuring better communication should improve compliance with therapies and prevent costly medical errors (11). Furthermore, facilitating access to primary care may diminish the use of emergency care, which is far more expensive (12). Thus, there are both ethical and financial reasons to develop medical structures adapted to the refugee population. Structures that would recognize and address the unique health care needs of refugees and the barriers they face upon resettlement.

One such innovation of the asylum care system was created at our medical center. Through the present article we (1) report the specific political context that motivated the elaboration of this facility; and (2) describe the establishment of our new health structure: the family consultation service of Lausanne, designed with the goal of ensuring optimal access to high quality care for Syrian families involved in the Swiss resettlement program.

## Political Context

Innovations are often created following specific political and societal pressures. In our case, the ongoing Syrian conflict was the trigger. It led the Swiss government to make changes to the asylum-related policy, which in turn required some adjustment at the medical care level.

The Syrian war, which started in 2011 has had considerable humanitarian consequences. As reported by the United Nations High Commissioner for Refugees [UNHCR; (13)] in 2018, this particularly violent and destructive conflict had led 6.7 million Syrians to seek safety beyond their borders. This statistic represents approximately one third of the Syrian population and does not account for the very large number of internally displaced persons. Furthermore, since 2014, Syria is the top source country for refugees worldwide (13).

In view of the situation, several European countries modified their asylum policies to facilitate the hosting of Syrian refugees. In Switzerland, the government decided to take part in the European relocation program wherein migrants are transported from the borders of Europe (especially Italy and Greece) to the host country, where they will have to go through the normal asylum process (14). Additionally, the federal council agreed to reintroduce the “resettlement policy” which had been stopped for 20 years, during which the asylum policy focused on other strategies. The reintroduction of resettlement was done through the launching of a pilot study (15).

The resettlement policy, established by the UNHCR, enables individuals to be selected and transferred from a country to where they initially escaped (often a country neighboring their homeland) to a third country that recognizes their refugee status, guarantees their safety, and grants them rights similar to resident nationals (16). Accordingly, one significant strength of the resettlement policy (compared to the European program) is that beneficiaries do not have to go through the host country's asylum procedures, as they are granted refugee status upon arrival. Through the article we differentiate between asylum seekers and refugees. Their status significantly differ as asylum seekers may face deportation to the country of origin, while refugees are in a more stable position.

The pilot project started in 2013. It was conducted in eight cantons, spanned 2 years and allowed the resettlement of 502 individuals, mainly families (14). The Swiss federal council provided 12 million CHFs to the different cantons to support the integration of the resettlement refugees. In addition, the cantons received 20,000 CHFs per refuge for specific integration measures (17). Considering its positive outcomes, such as the improvement of the occupational integration, the cost-effectiveness of the project was considered favorable (16), and in 2015 the Swiss government decided to launch a larger resettlement program, including all cantons, with the goal of hosting 3,000 additional Syrian refugees over a 3-year period (18). This decision required some logistical restructuring amongst different sectors, particularly health care.

## Health Care System Before Innovation

Switzerland is composed of 26 cantons, each of them being responsible for the organization of accommodation and healthcare of asylum seekers and refugees that are attributed to their territory. Some have specific dedicated health structure for this population, as other don't. Here we describe the case of the canton of Vaud in which the family consultation was implemented. This canton hosts ~10% of the migrants in Switzerland, which represented about 3,500 new requests of asylum in 2015, and 1,400 in 2019 (19). It should be noted that before this innovation, a specific medical system was already dedicated to asylum seekers but did not include refugees, as they were considered to have spent enough time in the country during the asylum procedure to be able to integrate the local health system. Asylum seekers, as well as refugees are both provided a health insurance since their arrival, that covers all necessary medical costs.

In the canton of Vaud, the medical care for adult asylum seekers is coordinated by the Migrant Care Unit at the University Center for Primary Care and Public Health (Unisanté) in collaboration with the Children's Hospital of Lausanne (HEL), which is in charge of all pediatric consultations. Unisanté has 850 employees, working in research, academic activities, disease prevention and clinical care. In 2018, it conducted 260,363 patient contacts, among which 19,233 involved the Migrant Care Unit (20). The HEL is a smaller structure with 450 employees and 64,547 consultations in 2018, among which 1,397 involved a translator.

For adult asylum seekers, Unisanté provides a medical evaluation on arrival, with a vaccination catch-up schedule, as well as serological check-ups for the hepatitis B virus and for HIV. For children, the HEL follows a similar procedure in addition to the administration of a Mantoux test to assess for tuberculosis. Unisanté and the HEL also cover the medical follow-up of these patients during the asylum process. This process has been accelerated since February 2019, however it may still take up to a year (21). As it is rather long, once asylum seekers are granted refugee status, they are considered to have had enough time and support to find their own family practitioner. Accordingly, they exit the asylum seeker medical system and enter the general medical system, just as any national. This is the reason why a specific medical system existed for asylum seekers but did not include refugees.

The reintroduction of the resettlement policy in 2013 challenged this procedure. Unlike asylum seekers, the individuals included in the resettlement project are delivered a residency permit upon arrival. As refugees, they exit the asylum seeker system and its related medical structure. Consequently, they lack the necessary time of adaptation to the Swiss medical system, which is problematic regarding health equity. For that reason, the Public Health Service of the canton of Vaud and the State Secretariat for Migration solicited the Migrant Care Unit of Unisanté to submit proposals on how to organize the medical care of this specific population, still in need of guidance but no longer tied to the asylum care system.

## The Family Consultation of Lausanne

A first possibility would have been to include the resettlement refugees in the asylum care system for a certain amount of time, before redirecting them toward the general medical system. However, one of the strengths of the Swiss resettlement program is that it almost exclusively concerns families. Being a refugee and a parent represents a double challenge (22). It means dealing with all the parenting tasks while adapting to a new environment and juggling between ones' own culture and the culture of the asylum country. Furthermore, families may have specific issues of concern. For these reasons, it seemed important to take into consideration this characteristic of the population (i.e., being a family) while searching for an optimized health care approach.

A novel solution-the creation of a family consultation service—was found. Its first aim was to provide resettlement families a similar care to that of asylum seekers and to ensure equity in terms of access and quality of health care.

**Figure 1:**
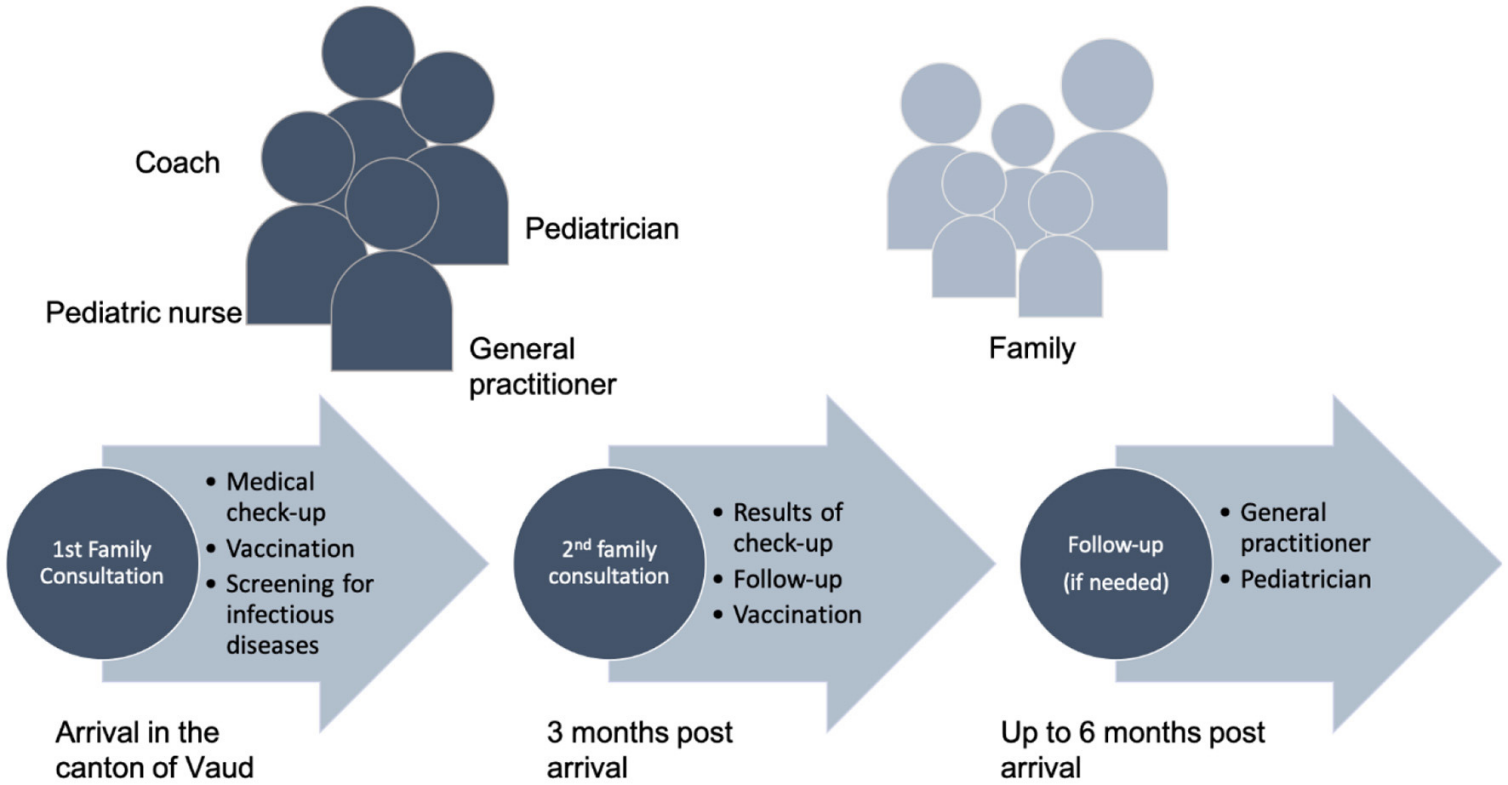
The family consultation.

The family consultation is organized as follows (Figure 1). It takes place in the HEL, where two consultation rooms are available once a week. The staff of the family consultation is composed of a pediatrician, a general practitioner and a pediatric nurse. The translation is done by coaches who are part of the resettlement program and are mandated to assist and support the families during their first 2 years in Switzerland. The coaches are native Arabic speakers and as such, also play a role in providing cultural support. It should be noted that the coaches benefit from the supervision of a transcultural psychiatrist.

The family consultation involves the initial medical evaluation (after arrival in the canton of Vaud), which is identical to the one delivered to asylum seekers (detailed above), as well as a follow up consultation 3 months after arrival. Half a day is devoted per family, as most families include several children and tend to have social and medical complexities (which are part of the selection criteria of the UNHCR) that require focused evaluation.

Each consultation starts with time dedicated to the whole family, during which the situation of the family and their common history is discussed in presence of the whole staff. Individual medical consultations and care follow, which include a detailed medical anamnesis, a complete status, blood tests and administration of the first vaccination shots if indicated. Finally, everyone meets again for a debriefing of the consultation during which the doctors explain what will happen next. The family members are also encouraged to ask questions and to share their thoughts about the consultation.

During the follow-up, families are given feedback regarding the tests that were performed at the initial medical evaluation, and additional vaccinations are administered as required. Regarding further medical needs (for example if one of the patients contracts an illness) the Migrant Care Unit of Unisanté oversees the adult consultations and the HEL the pediatric ones. When needed, patients are redirected toward a specialist.

The role of Unisanté and the HEL ends 6 months after the refugees' arrival in Switzerland. In the resettlement process, this should correspond to the period during which families move from group accommodations to individual housing. As families enter the general medical system, they are assisted in finding a general practitioner and a pediatrician close to home, either by their social assistant or by the medical team for special situations. A medical report is prepared summarizing the medical situation for the next practitioner.

Financially, the medical consultation provided by GPs and pediatricians are covered by the insurance of the patients. The salary of the nurse is paid for, by the Public Health Service of the canton of Vaud, as medical insurances don't cover much of the nurse's work. The interpreters are paid by the social services in charge of the refugees in the canton.

## Discussion

The family consultation service was created to ensure the delivery of adequate health care to Syrian families resettling in the Canton of Vaud, Switzerland. Indeed, the refugee status implies that resettlement families should directly enter the general medical system. However, this would represent multiple barriers in their access to health care, therefore jeopardizing health equity. Accordingly, by providing an adapted facility, our primary goal was achieved.

The family consultation structure also appears to have logistical advantages. As a single consultation is organized for the whole family, the welfare services in charge of the refugees only have to organize one transport to the hospital. It also simplifies the providing of an interpreter by removing travel times from a worksite to another. Furthermore, by conducting an interdisciplinary team consultation, it facilitates communication between the different medical personnel and allows a global insight of the families' situation. Having a wide perspective on the family system may represent an advantage all the more significant as the families included in the resettlement program are selected based on their social and medical complexities, often requiring time intensive consultations and care. Finally, having a single consultation for the whole family reduces redundancies and therefore accelerates the consultation process. Indeed, patients do not have to repeat their personal information (e.g., migratory journey) to each medical actor. This might also reduce trauma as families do not have to keep retelling their story.

Another advantage of the family consultation is that it may lower the health costs associated with caring for recently resettled refugee families. Indeed, it should reduce the number of unnecessary emergency consultations, as families are thoroughly followed and benefit from a better coordination of the different medical actors. This should be true even though the family consultation dedicates further time to the family unit compared to individual appointments. However, the financial advantage is only an assumption and should be verified through further research.

Families and staff seem to be satisfied by this care approach. Indeed, families appear to enjoy the fact of coming in all at once, and medical residents show interest for taking shifts in this consultation. Nevertheless, research is needed (and is currently in process) to adequately evaluate patients' perception and satisfaction of care provided by the family consultation.

These encouraging early findings were not achieved without overcoming certain difficulties. In particular, the coordination of two institutions, usually working independently and using different internal organizations required an adjustment period. Accordingly, effective communication was essential during the launching period.

If we adopt a larger perspective, it should be noted that healthier individuals have greater chances of succeeding at integration (23). Thus, a system such as the family consultation should be beneficial at the individual level but also at a larger level, for the host country. Indeed, healthy refugees represent new labor forces, which are much needed in several European countries that suffer from the falling of birth rates on the one hand, and of the rising of life expectancy on the other hand. As emphasized by The University College of London–Lancet Commission on Migration and Health, benefits of modern migration are intertwined with individuals' health (10). Furthermore, healthy refugees may also contribute to the development of their country of origin. Accordingly, implementing adjusted facilities such as the family consultation may have cascading positive effects.

## Next Steps

The family consultation was initially created for a 3-year period, just as the resettlement program. Fortunately, in 2019 the Swiss government confirmed that it would remain committed to the resettlement program and decided to enlarge it to countries other than Syria (24). This political decision therefore granted sustainability to the family consultation.

Regarding the expanding possibilities, at the country level, several Swiss cities have expressed their interest in implementing similar health structures. Furthermore, considering the multiple advantages of the family consultation, options were considered to enlarge its prerogatives. It appeared that it could be particularly beneficial for families issued from the undocumented immigrant populations, and in situations of family reunification (i.e., a part of the family first arrives to the host country and enrolls in an asylum process allowing the other family members to join them with a refugee status).

A final aspect that should be mentioned is research. The structure of the family consultation has the advantage of including both parents and children. This characteristic enables a systemic perspective, therefore representing an interesting basis to launch research projects. In line with this perspective, the family consultation supported the instigation of an ongoing longitudinal study on the physical and mental health of Syrian refugee families (25).

## Conclusion

The challenges of providing effective and timely health care to the growing and diversified population of refugees have been increasingly recognized (26). Through the present article we described the implementation of an innovative health structure dedicated to the care of Syrian refugees in Switzerland.

The family consultation service presents an interesting and novel approach to respond to the social and medical needs of resettlement refugees. As with any new clinical service, it implied logistical adjustments, but the efforts required appear to have been fruitful. Accordingly, it provided an adapted facility to 76 families representing 329 individuals between 2017 and 2021. The family consultation shows practical and organizational advantages, for the medical team and for the patients: first, the presence of the whole medical team allows direct exchanges between the different actors and optimizes communication; second, it allows a broad perspective on the family system; third, it accelerates the process by reducing redundancies (one file per family). Hence, it seems to enhance the staff and families' satisfaction, while ensuring high quality care. The family consultation also seems to be appropriate to other populations in situation of vulnerability, allowing a possible generalization of this medical approach.

## Data Availability Statement

The original contributions presented in the study are included in the article/Supplementary Material, further inquiries can be directed to the corresponding author.

## Author Contributions

JB, NE, and MM wrote sections of the manuscript. All authors contributed to manuscript revision, read, and approved the submitted version.

## Conflict of Interest

The authors declare that the research was conducted in the absence of any commercial or financial relationships that could be construed as a potential conflict of interest.

## Publisher's Note

All claims expressed in this article are solely those of the authors and do not necessarily represent those of their affiliated organizations, or those of the publisher, the editors and the reviewers. Any product that may be evaluated in this article, or claim that may be made by its manufacturer, is not guaranteed or endorsed by the publisher.
