# Supplementary material for: Medical Facilities for Refugees in Europe: Creating a Consultation for Resettled Syrian Families
Source: Front Med (Lausanne). 2021 Nov 26;8:728878. doi: 10.3389/fmed.2021.728878 (PMC8662520; doi:10.3389/fmed.2021.728878)
Supplement: Supplementary file 1 [file Table_1.PDF]

ANNEX 1

Country	Total number of refugees (end-2020)[1]	Refugees to 1000 inhabitants[1]	Number of asylum seekers (end-2020)[1]	Asylum seekers to 1000 inhabitan	MIPEX general index[2]	MIPEX health index[2]	MIPEX International policies comparison[2]	MIPEX commentary on health policy[2]
**Switzerland**	115868	13.39	6547	0.76	50	83	The integration policies of Switzerland score a little below the average neighbouring countries or OECD countries.<br>Switzerland has some of the most restrictive policies in Western Europe and non-EU citizens are less likely to reunite with their family, enjoy a secure status or become a full citizen.	The Programme on Migration and Health is considered as world-leading for its innovations in the healthcare system.<br>All migrants in Switzerland benefit from a basic medical insurance for.
**France**	436100	6.68	118137	1.81	56	65	The score of integration policies in France is comparable to other Western European and OECD countries.<br>MIPEX considers it's approcach to integration as "Temporary Integration", like Italy, Germany, the Netherlands and the United Kingdom: "While non-EU citizens can benefit from basic rights and some support for equal opportunities, but not the long-term security they need to settle permanently, invest in integration and participate as full citizens."	Health services in France are inclusive and accessible, but does relatively little to address migrant patients' specific health needs<br>Regarding healthcare, migrants have the same rights legal residents and citizens.
**Germany**	1210636	14.45	243156	2.90	58	63	Migration policies have improved over the past decade.<br>Facing many arrivals in 2015/16, Germany improved the implementation of the existing system, with packages of measures and funding.<br>Non-EU immigrants face greater delays, uncertainty and obstacles than in nearly all MIPEX countries, similar only to neighbouring Austria, Denmark and Switzerland for a long term integration.<br>MIPEX considers the integration policies in Germany as "less comprehensive when compared to other Nordic countries, neighbouring Belgium and Luxembourg or the traditional destination countries".	Germany's policies lack a comprehensive approach and limit entitlements and access for undocumented migrants and asylum-seekers, even if some improvement is noted in responding to migrants' specific needs.
**Italy**	128033	2.12	53901	0.89	58	79	Italy scores 58/100, higher than the average MIPEX country (50) and slightly above-average among EU and Western European (EU15) / OECD countries<br>MIPEX consider it's approcach to integration as "Temporary Integration", like France, Germany, the Netherlands and the United Kingdom: "While non-EU citizens can benefit from basic rights and some support for equal opportunities, but not the long-term security they need to settle permanently, invest in integration and participate as full citizens."<br>Spain present a more comprehensive integration approach.	Legal migrants and asylum-seekers can enrol in the National Health Service, but the documentation required to access healthcare can be complicated for them.
**Spain**	103679	2.22	103385	2.21	60	81	Immigrants to Spain can benefit from many of the same basic rights as Spanish citizens. Integration policies don't entirely secure equal opportunities for non-EU citizens.<br>MIPEX considers that Spain has a more favourable approach than Italy and France, However, like all new European destination countries, it has less favourable policies than non-EU destination countries.	Some administrative barriers remain for immigrants to access healthcare, but they can benefit from responsive services and their rights are well explained.
**Portugal**	2445	0.24	1154	0.11	81	65	Portugal is one of the leading MIPEX countries, along some Nordics and traditional destination countries.<br>Portugal's integration scores in 2019 were above average in all policy areas except migrant health.	Portugal scores lower than average on health policies. Healthcare access and information has slowly improved by 2019.
**Sweden**	248425	24.6	18621	1.84	86	83	Sweden's approach to integration is similar to those of Canada, Finland and Portugal, and is more inclusive than those of the other Nordic countries. They guarantee equal rights, multiple opportunities and long-term security for immigrants.<br><br>Sweden's policies are considered as more ambitious than other countries by MIPEX and are more effective to meet immigrants in need.	On MIPEX, Sweden's health policies rank #2 just like Switzerland and New Zealand. They entitle legal immigrants, undocumented immigrants to almost the same level of healthcare as Swedish citizens.<br>However, rejected asylum-seekers lose their so-called LMA card, which creates difficulties for them to follow up on previous care. Immigrants also receive other forms of support in Sweden, such as interpretation.
**United Kingdom (before Brexit)**	132349	1.95	77245	1.14	56	75	British integration policies scores are above the average EU country and like the Western Europea.<br><br>MIPEX considers it's approcach to integration as "Temporary Integration", like France, Germany, the Netherlands and Italy: "While non-EU citizens can benefit from basic rights and some support for equal opportunities, but not the long-term security they need to settle permanently, invest in integration and participate as full citizens."	Immigrants receive some support to access health care, like information, provision of interpreters. However, because of legal restrictions and administrative obstacles, they face slightly weak entitlements to free NHS care. The Immigration Law from 2014 restricts migrants' access to free NHS hospital treatments and creates problems of discretion and documentation for migrants.

**USA**	340881	1.03	997 989	3.02	73	79	During the past four years, the US federal government has exacerbated the costs, delays and insecurity for immigrants. These changes defer many from the American dream of citizenship, a secure family, and a good job and have impacted the traditional gift for integration of the US.<br>immigrants in Canada, New Zealand, Portugal, Finland and Sweden, enjoy more equal opportunities and long-term security than in the US.	Immigrants who can meet the complicated entitlement rules are offered accessible and responsive healthcare services in the US.
**Australia**	57451	2.25	80803	3.16	65	79	Since 2014, immigrants in Australia now face more barriers to basic rights and equal opportunities, especially in labour market participation and to obtain permanent residence.<br>Despite these restrictive policies, Australia benefits from its well-developed comprehensive approach to integration.<br>Australia figuress in the MIPEX 'Top Ten' but score below the Canada, New Zealand and US but above Western European and OECD countries.	The healthcare system in Australis is well adapted to meed migrants specific needs. Australia rank in the top ten for health policies, but immigrants still face some obstacles in accessing healthcare depending on their legal status.
**Canada**	109264	2.9	85352	2.27	80	73	Canada scores 80 on the MIPEX 100-point scale, well above the MIPEX average of 50, and in the MIPEX 'Top Ten' with it's comprehensive approach.<br>Policies in most areas are slightly more favourable than in Australia, New Zealand and the US.	Canada's healthcare services are responsive and become more accessible for migrants, even if some obsacles still remain.

MIPEX: Migrant Integration Policy Index

1. Numbers from: https://www.unhcr.org/2020-global-trends-annex.xlsx
2. Data from: https://www.mipex.eu
